# Supplementary material for: Towards capability-adjusted life years in public health and social welfare: Results from a Swedish survey on ranking capabilities
Source: PLoS One. 2020 Dec 1;15(12):e0242699. doi: 10.1371/journal.pone.0242699 (PMC7707509; doi:10.1371/journal.pone.0242699)
Supplement: S1 Questionnaire — (DOCX) [file pone.0242699.s001.docx]

# S1. Questionnaire.

The original wording of the questionnaire used, in Swedish and the English translation. The original survey was implemented in Surveymonkey and is reproduced below as closely as possible. The translation was not done by a professional translator and only serves to document the survey methods.

| Original (Swedish) | Translated English |
| --- | --- |
| **Question 1:** | |
| Samtycke: Genom att svara Ja på nedanstående fråga samtycker jag till att delta i studien. Jag har tagit del av den information som medverkande forskare har skickat ut.  Samtycker Du till att frivilligt delta i studien? | Consent: By answering Yes to the question below I consent to participate in the study. I received the information that the researchers sent out.  Do you consent to participate voluntarily in the study? |
| *Answer options:* | |
| Ja, Nei | Yes, No |
|  | |
| **Question 2:** | |
| Rangordna dessa möjligheter/förmågor med en UNIK siffra från 1 (VIKTIGAST) till 10 (MINST VIKTIG). | Rank these opportunities/capabilities with a UNIQUE number from 1 (MOST IMPORTANT) to 10 (LEAST IMPORTANT) |
| *Answer option: Numeric text field* | |
| **Tidsutrymme:** Att ha denna möjlighet/förmåga innebär att ”för det mesta uppleva balans mellan det man bör göra (arbete och andra åtaganden) och det man vill göra (umgänge, kultur, hobby, osv.)” | **Timebalance:** To have this opportunity/capability implies to “mostly experience balance between what one needs to do (work and other) one what one wants to do (socialising, culture, hobby, etc.)” |
| *Answer option: Numeric text field* | |
| **Ekonomiska resurser:** Att ha denna möjlighet/förmåga innebär att ”ha en ekonomi (lön, annan inkomst eller besparingar) som tillåter en att leva på ett sätt som man i allt väsentligt vill.” | **Financial situation:** To have this opportunity/capability implies to “have a financial situation (salary, other income or savings) that mostly allows one to live in a way one wants.” |
| *Answer option: Numeric text field* | |
| **Hälsa:** Att ha denna möjlighet/förmåga innebär att ”ha ett bra allmänt hälsotillstånd (psykiskt och fysiskt) som mycket sällan begränsar ens möjlighet att arbeta eller ägna sig åt det man vill.” | **Health:** To have this opportunity/capability implies to “have a good general state of health (mentally and physically) that very seldom limits one’s possibility to work or do what one wants to do.” |
| *Answer option: Numeric text field* | |
| **Politiska resurser och medborgerliga rättigheter:** Att ha denna möjlighet/förmåga innebär att ”kunna bilda sig åsikter om små och stora frågor, kunna framföra dessa (vallokal, förening, myndighet, media, osv.) och att då bli bemött med respekt.” | **Political resources and civil rights:** To have this opportunity/capability implies to “be able to form opinions on big and small topics, to represent those opinions (ballot, association, authorities, media, etc.) while being met with respect” |
| **Kunskap och färdigheter**: Att ha denna möjlighet/förmåga innebär att ”ha den utbildning, erfarenhet och skicklighet som krävs för att i stort sett kunna arbeta med och ägna sig åt det man vill.” | **Knowledge and skills**: To have this opportunity/capability implies to “mostly have the education, experience and skill that is needed to be able to work and do what one wants.” |
| *Answer option: Numeric text field* | |
| **Livsmiljö:** Att ha denna möjlighet/förmåga innebär att ”leva i ett närsamhälle (människor, bebyggelse, parker/natur, transporter, affärer, etc.) som man i allt väsentligt är nöjd med.” | **Living environment:** To have this opportunity/capability implies to “live in an environment (people, buildings, nature, infrastructure, shops, etc.) that one is mostly satisfied with.” |
| *Answer option: Numeric text field* | |
| **Sysselsättning:** Att ha denna möjlighet/förmåga innebär att ”ha ett arbete eller annan sysselsättning (studier, praktik, hushållsarbete, vård av anhörig, etc.) som man i stort sett är nöjd med.” | **Occupation:** To have this opportunity/capability implies to “have work or other occupation (studies, internship, domestic work, giving care to relatives, etc.) that one is mostly happy with.” |
| *Answer option: Numeric text field* | |
| **Sociala relationer:** Att ha denna möjlighet/förmåga innebär att ”ha tillgång till nära relationer (familj, vänner eller bekanta) som bidrar till att man trivs och utvecklas, och som ger en råd och stöd när man behöver.” | **Social relations:** To have this opportunity/capability implies to “have access to close relations (family, friends and acquaintances) that contribute that support one’s general well-being and development, and provide advice and support in case of need.” |
| *Answer option: Numeric text field* | |
| **Säkerhet till liv och egendom:** Att ha denna möjlighet/förmåga innebär att ”i princip aldrig känna sig rädd för att drabbas av inbrott, skadegörelse, våld av olika slag eller hot om våld, varken i hemmet eller på annan plats.” | **Safety for life and property:** To have this opportunity/capability implies to “in principle never having to worry about burglary, damages, harm, different kinds of violence or threats of violence, neither at home or elsewhere.” |
| *Answer option: Numeric text field* | |
| **Bostad:** Att ha denna möjlighet/förmåga innebär att ”ha ett boende som man är trygg med när det gäller kontrakt eller ägande, och nöjd med när det gäller funktion, utseende och läge”. | **Accommodation:** To have this opportunity/capability implies to “have a permanent accommodation that is stable in terms of rental agreement or ownership, and satisfied with in terms of functionality, appearance, and location”. |
|  | |
| **Question 3:** | |
| Kön | Gender |
| *Answer options* | |
| Kvinna, Man, eller lämna tom | Man, woman, or leave empty |
|  | |
| **Question 4:** | |
| Ålder | Age |
| *Answer options:* | |
| Under 30, 30-39, 40-49, 50-59, 60 och över | Under 30, 30-39, 40-49, 50-59, 60 and over |
|  | |
| **Question 5:** | |
| Forskningsområde | Area of research |
| *Answer option: Free text* | |
|  | |
| **Question 6:** | |
| Eventuella reflektioner | Eventual reflections |
| *Answer option: Free text* | |
